# Anti-inflammatory and Antioxidant Activity of Peptides From Ethanol-Soluble Hydrolysates of Sturgeon (*Acipenser schrenckii*) Cartilage

**DOI:** 10.3389/fnut.2021.689648

**Published:** 2021-06-11

**Authors:** Li Yuan, Qian Chu, Xiaoyun Wu, Bei Yang, Wei Zhang, Wengang Jin, Ruichang Gao

**Affiliations:** ^1^School of Food and Biological Engineering, Jiangsu University, Zhenjiang, China; ^2^Bio-Resources Key Laboratory of Shaanxi Province, School of Biological Science and Engineering, Shaanxi University of Technology, Hanzhong, China

**Keywords:** cartilage, anti-inflammatory, ethanol extraction, Sephadex G-15, MAPK

## Abstract

Research has shown that cartilage containing chondroitin sulfate and protein presents versatile bioactivities. Chondroitin sulfate in cartilage is beneficial to activate the immune system while the protein/peptide has not been fully understood. The current study investigated the antioxidant and anti-inflammatory properties of ethanol-soluble hydrolysates of sturgeon cartilage (ESCH) prepared through hot-pressure, enzymatic hydrolysis and ethanol extraction. UV spectrum, IR and agarose gel electrophoresis results suggested the successful exclusion of chondroitin sulfate from peptides. Nitric oxide (NO) floods in cells activated by inflammation. It was inhibited when administrated with ESCH. To further explain the observed anti-inflammatory activity, ESCH was separated with Sephadex G-15 into 3 components, among which F3 showed a higher NO inhibition rate and significantly reduced the production of the proinflammatory cytokine IL-6. In addition, the yield of IL-10 increased. Western blotting suggested that F3 downregulated the NO content and IL-6 level by suppressing Mitogen-activated protein kinases (MAPK) channels. Moreover, both ESCH and F3 showed DPPH and ABTS free radical scavenging abilities which was possibly related to the anti-inflammatory property. These results indicated that ESCH behaved anti-inflammatory and antioxidant activities. Cartilage may be a good source to produce anti-inflammatory peptides.

## Introduction

Innate immunity, also known as natural immunity, is different from specific immunity and mainly represents the first line of defense against infection through anatomic, physiologic, cell or inflammatory components. Macrophages and neutrophils of the innate immune system are dominant in phagocytosis (1). Because RAW264.7 macrophage cells are easy to reproduce, highly efficient in DNA transfection and sensitive to RNA interference, they are usually used to study the mechanism of bioactive substances in immunity (2–4) and inflammation (5, 6). During the inflammatory response, cells will produce a large number of inflammatory mediators and inflammatory cytokines, such as NO, IL-6 TNF-α (6). NO has a variety of physiological and pathological functions. It not only has a wide range of synthesis and distribution but also has a variety of mechanisms, playing an important role in inflammation and immunity (7). Many studies regarded NO as an important index of immune regulation and anti-inflammatory activity (3, 8–11). Cytokines play an important role in the regulation of the immune response, inflammation and homeostasis (3, 12). TNF-α is one of the cytokines participating in the inflammatory response and mediates a variety of biological processes, such as antitumour and immune regulation (13). IL-6 plays an important role in regulating cell growth and differentiation, immune function, hematopoietic function and anti-inflammatory function (14). Therefore, reversing the excessive production of IL-6 and TNF-α is considered to be an effective method for the treatment of inflammatory diseases (15, 16). As anti-inflammatory cytokine, IL-10 can block the activity of Th17 cells and inhibit the inflammatory reaction by reducing the synthesis of proinflammatory factors (9, 16). And TGF-β contributes to the tissue repair (17).

In recent years, bioactive peptides derived from discarded protein have been widely reported (18). Frames containing bone and cartilage are classified as animal by-products, and they are usually processed as low-market-value products, such as animal feed, fish meal and fertilizer (19, 20). Studies have demonstrated peptides released from cartilage possess multifarious biological activities, including immunity, antioxidation, anti-hyperuricemia and anti-osteoporosis (21–25). Protein and chondroitin sulfate are the main components in cartilage (26). The former shows the promise to induce inflammatory cytokines (27), and the latter is indicated to play an important role in immune stimulation and anti-inflammatory (26, 28). However, whether peptides from sturgeon cartilage possess anti-inflammatory activity do not been determined and its underlying mechanisms have not been resolved.

In the present study, ESCH was prepared from sturgeon cartilage through hot-pressure, enzymolysis and ethanol extraction. Study reported that the antioxidant system may affect anti-inflammatory effects through the MAPK/PI3K-AKT/TNF/NF-κB/TCR/TLR signal pathway (29), suggesting the connection between the antioxidant system and the immune stimulation response (30). The collected ESCH was further utilized to investigate the anti-inflammatory and antioxidant activities and the specific molecular mechanism in LPS-induced RAW264.7 macrophages.

## Materials and Methods

### Materials

Sturgeon (*Acipenser Schrenckii*) cartilage by-product was rendered from Quzhou Xunlong Aquatic Products Sci-tech Development Co., Ltd. (Quzhou, China). Papain (papaya latex), pancreatin (porcine pancreas) were brought from Sinopharm Chemical Reagent Co., Ltd., (Shanghai, China). Fetal bovine serum (FBS), Dulbecco's modified eagle medium (DMEM) were obtained from Biological Industries (Beit Haemek, Israel). Total NO Assay Kit, Lipopolysaccharide (LPS, TLR4 activator) were purchased from Beyotime Biotechnology (Shanghai, China). Cell Counting Kit-8 was from ApexBio technology (Houston, USA). Inflammatory cytokines kit including Mouse IL-6 ELISA kit, Mouse TGF-β ELISA kit and Mouse IL-10 ELISA kit were obtained from FCMAchondroitin sulfate (Nanjing, China). MAPK Family Antibody Sampler Kit was purchased from Cell Signaling Technology (Beverly, MA, USA). 2,2-diphenyl-1-picrylhydrazyl (DPPH), 2,2-azino-bis(3-ethylbenzthiazoline)-6-sulfonic acid (ABTS) were both obtained from Aladdin Industrial Corporation (California, USA).

### Preparation of ESCH

The by-product of cartilage was thawed at 4°C. For reducing impurities as much as possible, the cartilage was boiled in boiling water for 20 min to remove the spinal cord, meat, lipid and fascia on the surface. After separating the clean cartilage, the cartilage was cut into small particles at 4°C with a chopper, mixed them well. To avoid repeated freezing and thawing, the small cartilage particles were packed separately and stored at −20°C before use.

The preparation of ESCH was referred to Shen et al. (31) with some modifications. Briefly, hot-pressure method was used to extract protein. Cartilage was mixed with distilled water at 1:2.5 (w/v) to ensure that cartilage was immersed in distilled water. After liquefying for 90 min at 120°C, the residual solid was homogenized to obtain the hot-pressed extract (HPE). The protein content of HPE was adjusted with distilled water to 5% (w/w). The enzymatic hydrolysis process was as follows: 0.65% of trypsin at 37°C, pH 7.0 for 2 h, 0.5% of papain at 60°C, pH 7.0 for another 2 h. The hydrolysate was heated at 90°C for 15 min and centrifuged at 4°C 10,000 rpm for 30 min to remove oil and insoluble substances. The supernatant was lyophilized for 48 h to obtain the sturgeon cartilage hydrolysate (SCH). For ethanol extraction, SCH was dissolved in distilled water (50 mg/mL). Final concentration of ethanol in this solution was 85%. The mixture was stirred at 4°C for 24 h and then centrifuged at 4°C 10,000 rpm for 30 min. Supernatant was collected to rotary evaporation. The ethanol-soluble sturgeon cartilage hydrolysate (ESCH) was finally obtained.

### Basic Composition and Liquefaction Rate of Sturgeon Cartilage

The basic composition and liquefaction rate was determined (31, 32).

### Agarose-Gel Electrophoresis

An instrument JY-SPCT (JUNYI Electrophoresis Company, Beijing, China) was used to execute the agarose-gel electrophoresis. Methods refer to previous studies with modification (31–33). One percent agarose gel was prepared in 0.04 M barium acetate buffer. The samples and chondroitin sulfate standard were run in 1,2-diaminopropane at 100 mA for 120 min. After electrophoresis, gels were soaked in cetyltrimethylammonium chloride for 120 min.

### UV Spectrum and IR Spectra Analysis

The ESCH was dissolved in deionized water and adjusted to 1 mg/mL. The UV spectrum was measured at 190–400 nm by UV spectrophotometer. FT-IR (Fourier Transform Infrared Spectroscopy) was used to determine the IR of sample in the frequency range of 4,000–500 cm^−1^ (33).

### Determination of Degree of Hydrolysis and Molecular Weight Distribution

The hydrolysis degree (DH) of ESCH was determined by OPA method (34). The sample solution of 400 μL was added to 3 mL OPA reagent, mixed for 5 s and stood for 2 min. The absorbance value at 340 nm was measured immediately. The molecular weight of ESCH was determined according Irvine and Shaw (35). In short, TSK gel 2000swxl column (7.8 × 300 mm) was used. The mobile phase was acetonitrile/water/trifluoroacetic acid (45:55:0.1, v/v). The standard molecular weight solutions of cytochrome c (MW 2400), bacillase (MW 450), Gln-Gln-Tyr-Arg (MW 451) and Gln-Gln-Gln (MW 189) were dissolved in mobile phase to prepare a standard molecular weight solution (0.2 mg/mL). ESCH was prepared into 10 mg/mL. The whole process was monitored at 220 nm.

### Quantification of Amino Acids

ESCH was hydrolyzed with 6 M HCl at 110°C, and then removed for filtration after 18 h and transferred to a 50 mL volumetric flask. Take 3 mL of hydrolysate and dry it completely in vacuum at 45°C. It was re-dissolved and dried again. The processed sample was finally dissolved in sample diluent, and then analyzed by SYKAM-433D automatic amino acid analyzer. The amino acid content was expressed as residue number/1,000 residues.

### Antioxidant Activity

The antioxidant activity of ESCH and F3 were evaluated from the scavenging ability of DPPH and ABTS^**+.**^(36). In short, the sample dissolved in 1 mL deionized water was mixed with 1 mL of DPPH solution (0.1 mM, ethanol), then stood still for 30 min. Absorbance was detected at 517 nm. Mixture of 200 μL sample solution and 2 mL of ABTS working solution [*A*_(730)_ = 0.70 ± 0.02] was incubated for 20 min. Absorbance was detected at 730 nm.

### Gel Filtration Chromatography

According to the molecular weight distribution of ESCH, Sephadex G-15 (Φ 1.2 × 60 cm, GE Healthcare) was used for separation. The flow rate and loading volume were adjusted to 0.8 mL/min and 1 mL (50 mg/mL).

### NO and Inflammatory Cytokine Production in LPS-Induced RAW264.7 Cells

#### Cell Culture and Cell Viability

RAW264.7 macrophages were cultured in DMEM containing 10% FBS and 1% penicillin and streptomycin solution in a 37°C incubator containing 5% CO_2_. When the cells reached 80–90% confluence, they were gently blown with fresh medium and passaged at a ratio of 1:3.

The cell density was adjusted to 5 × 10^5^ cells/mL and added to a 96-well plate for 24 h. After that, fresh media (0, 12.5, 50, 200, 400, and 800 μg/mL) containing ESCH was added for another 24 h. Then, 10 μL CCK-8 was added. Cell viability was determined according to the method described in the CCK-8 kit. The absorbance was measured at 450 nm and calculated as follows:

$$\begin{array}{l} \text{Cells Viability}\left(\%\right)=\frac{OD_{ESCH}-OD_{blank}}{OD_{NC}-OD_{blank}}\times 100\% \end{array}$$

ESCH: with ESCH treatment; blank: medium only; NC: cells without ESCH treatment.

#### Determination of NO Content

The release of NO was used as the screening index of anti-inflammatory activity of ESCH and its separated components. The cells were added to 96 well plate for culture. Fresh media containing different doses of ESCH were added for 2 h pre-treatment, and then induced by LPS (2 μg/mL) for 22 h. The operation was carried out according to the NO reagent method. Absorbance value was measured at 540 nm.

#### Enzyme Linked Immunosorbent Assay

The productions of IL-6, TNF-α, IL-10 and TGF-β in cell culture supernatant were measured by the ELISA kits.

### Western Blotting

Western Blotting procedures were conducted by previous study with slightly modification (37). Cells after treatment were collected by RIPA lysate containing protease inhibitors and phosphatase inhibitors. Total protein of different groups was normalized by BCA protein assay kit.

### Peptide Sequence Identification

The peptides sequence of F3 was identified by Bio-Tech Pack Technology Company Ltd. (Beijing, China).

### Statistical Analysis

Statistical analysis was calculated using SPSS (version 22.0; Chicago, IL, USA). All data were expressed as means ± standard deviation (SD) (*n* = 3). Differences between the means of each group were assessed by one-way ANOVA (Tukey's multiple-range test). *p* < 0.05 was considered statistically significant.

## Results

### Basic Composition and Liquefaction Rate of Sturgeon Cartilage

The moisture, protein and ash contents of sturgeon cartilage particles were 82.21, 9.38, and 6.17%, respectively. Under the condition of 120°C, 90 min, the liquefaction rate of sturgeon cartilage was up to 98.10%.

### DH and Molecular Weight Distribution

DH was determined through OPA method. The DH of cartilage hydrolysates obtained in this research was 21.02 ± 0.45%. According to Table 1, the content of low molecular weight peptide in sturgeon cartilage was 94.71%. Among them, 24.83% of the polypeptides were 500 Da−1.0 kDa, 60.31% were 180 Da−500 Da, 9.57% were below 180 Da, and the average molecular weight was 426 Da.

**Table 1:** Molecular weight distribution of ESCH.

**Molecular weight**	**Percentage of peak area**	**Weight average molecular weight**
>10,000	0.01	12,445
10,000–5,000	0.05	6,827
5,000–3,000	0.19	3,625
3,000–2,000	0.65	2,361
2,000–1,000	4.40	1,343
1,000–500	24.83	678
500–180	60.31	272
<180	9.57	92

### Agarose-Gel Electrophoresis, UV and FT-IR Analysis

**Figure 1:**
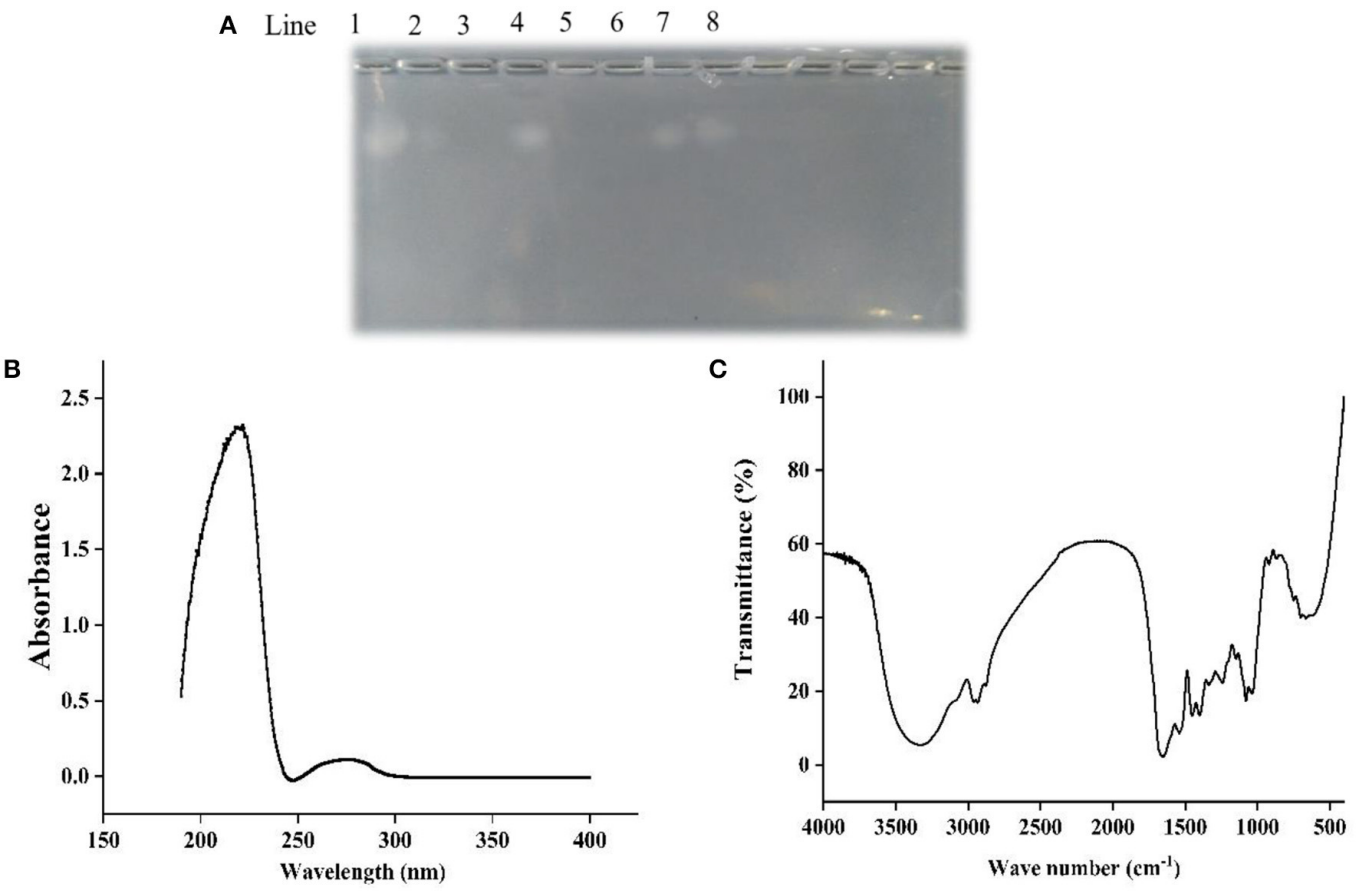
Agarose-gel electrophoresis **(A)**, UV **(B)**, and FT-IR **(C)** of Echondroitin sulfate. Line 1 was chondroitin sulfate standard, line 2 and 3 were 75 and 85% ethanol extracts of double enzyme hydrolysate, line 4 and 5 were 75 and 85% ethanol extracts of papain hydrolysate, line 6 and 7 were <10 kDa and more than 10 kDa components of double enzyme hydrolysate, and line 8 were 85% ethanol precipitation components of double enzyme hydrolysate.

Figure 1A showed that chondroitin sulfate could be observed both in the ethanol extract of papain and double enzyme hydrolysis under 75% ethanol extraction condition, although the band of the latter was shallower than that of the former, which suggested that the residue of chondroitin sulfate was lower. When the ethanol concentration reached 85%, there was no chondroitin sulfate residue. Ultrafiltration was used to determine the approximately molecular weights of chondroitin sulfate. The electrophoresis results showed that a band of chondroitin sulfate occurred in the components larger than 10 kDa rather than the components smaller than 10 kDa, revealing the molecular weights of chondroitin sulfate was higher than 10 kDa.

In the UV spectrum of ESCH (Figure 1B), there was a strong absorption peak near 220 nm and a weak absorption peak near 280 nm, which indicated that there were polypeptides with benzene ring structures, such as phenylalanine, tryptophan and tyrosine.

As shown in Figure 1C, ESCH showed amide A and amide B peaks at 3334.86 and 2960.25 cm^−1^, represented N-H stretching and =C-H vibration. The amide I and amide II bands were 1658.99 and 1539.91 cm^−1^, resulted from the C=O stretching vibration, C-N stretching vibration and N-H in-plane bending of the peptide bond. In the infrared spectrum of ESCH, there were characteristic absorption peaks at 921.34, 1242.92, and 867.34 cm^−1^, which suggested that there might be carbohydrates in the extracted ESCH.

### Amino Acid Analysis

The amino acid content analysis of ESCH is shown in Table 2. The amino acid composition of ESCH showed that it contained a high content of glycine (275.52 residues/1,000 residues), followed by proline (126.19 residues/1,000 residues) and alanine (117.44 residues/1,000 residues). Aromatic amino acids content including phenylalanine and tyrosine was up to 40.86 residues/1,000 residues.

**Table 2:** Amino acid analysis of ESCH.

**Amino acid**	**Contents (residues/1,000 residues)**
Asp	31.65 ± 0.24
Thr	32.08 ± 0.22
Ser	35.76 ± 0.14
Glu	74.52 ± 0.89
Gly	275.52 ± 0.55
Ala	117.44 ± 0.82
Val	42.97 ± 0.96
Met	17.30 ± 0.01
Ile	30.17 ± 0.17
Leu	62.24 ± 0.23
Tyr	13.50 ± 0.36
Phe	27.36 ± 0.23
His	5.55 ± 0.03
Lys	28.88 ± 0.16
Arg	19.79 ± 0.63
Pro	126.19 ± 1.10
Hyp	58.08 ± 5.12
Hydrophobic AAs	470.26 ± 2.47

In addition, the contents of histidine (5.55 residues/1,000 residues), tyrosine (13.50 residues/1,000 residues), methionine (17.30 residues/1,000 residues) and arginine (19.79 residues/1,000 residues) were lower than others. In ESCH, the amount of hydrophobic amino acids was close to half of the total amino acids.

### Cell Viability and NO Release of ESCH

**Figure 2:**
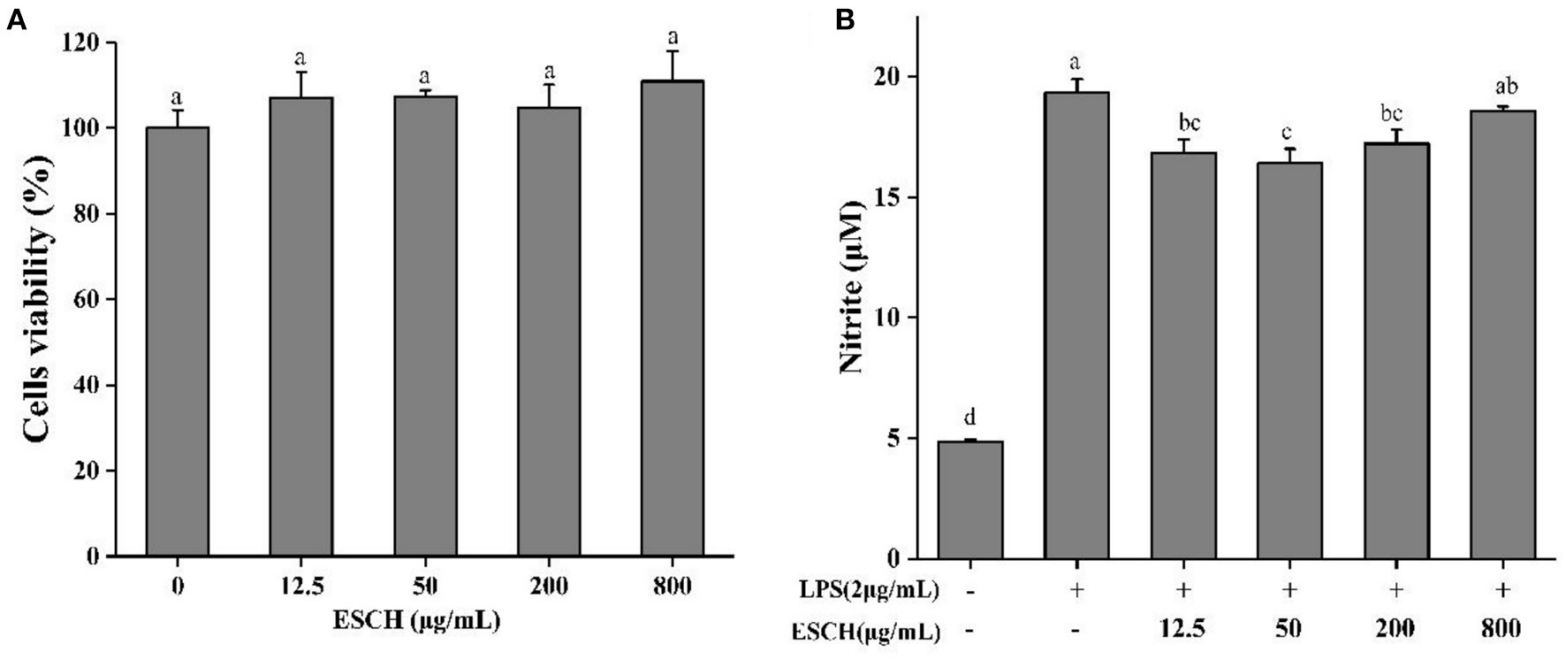
Cell viability **(A)** and NO release **(B)** of ESCH in RAW264.7 macrophage. The results were expressed as means ± SD (*n* = 3), where lowercase letters indicate significantly different at *p* < 0.05.

CCK-8 was used to determine the effect of ESCH on the activity of RAW264.7 cells before evaluating the anti-inflammatory activity of ESCH *in vitro*. Figure 2A shows that 3.125–800 μg/mL ESCH did not show a significant toxic effect on RAW264.7 macrophages within 24 h. Therefore, a nontoxic dose of ESCH was used to evaluate its anti-inflammatory potential. As shown in Figure 2B, the inhibition rates of ESCH at concentrations of 12.5, 50, and 200 μg/mL on NO release from macrophages were 12.93, 15.19, and 11.03%, respectively (*p* < 0.001). There was a “U”-type dose-response relationship, and the best inhibitory effect was at a concentration of 50 μg/mL.

### Sephadex G-15 Separation and NO Determination

**Figure 3:**
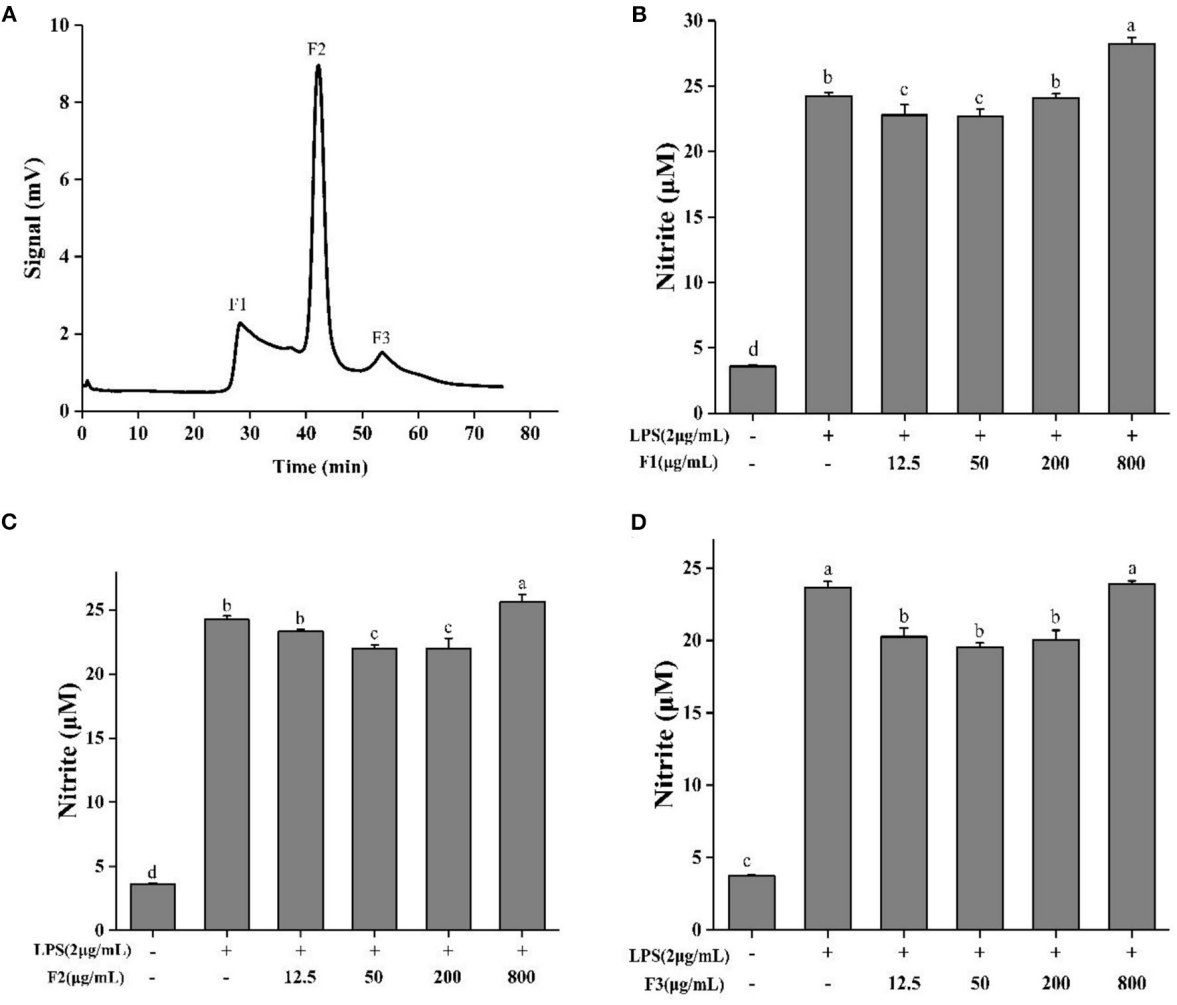
Sephadex G-15 separation **(A)** of ESCH and NO determination of different components named F1, F2, F3 **(B–D)** in RAW264.7 macrophage. The results were expressed as means ± SD (*n* = 3), where lowercase letters indicate significantly different at *p* < 0.05.

ESCH was separated by Sephadex G-15 (Φ 1.2 × 60 cm). According to different elution times, three components were obtained, named F1, F2, and F3 (Figure 3A). The inhibition rates of NO were 5.95–6.37%, 6.28–9.38%, and 14.39–22.16% (Figures 3B–D). The effect of F3 was more obvious, indicating that F3 may have higher anti-inflammatory activity. This result may be due to the average molecular weight of F3, which is smaller than that of the other two components.

### DPPH and ABTS Scavenge Ability

**Figure 4:**
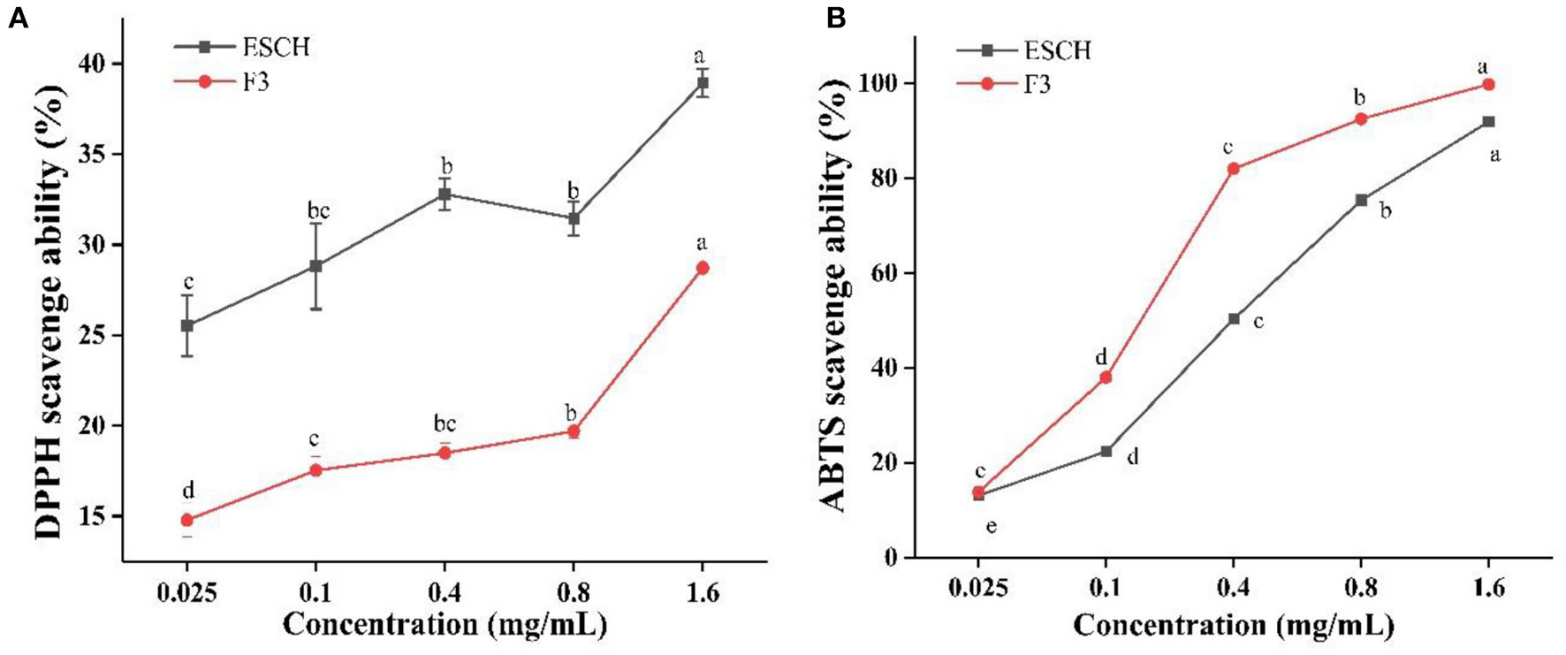
DPPH **(A)** and ABTS **(B)** scavenge ability of ESCH and F3. The results were expressed as means ± SD (*n* = 3), where lowercase letters indicate significantly different at *p* < 0.05.

Antioxidant activity was assessed by free radical scavenging (Figure 4). At the highest concentration of 1.6 mg/mL, F3 showed a good *in vitro* antioxidant capacity: 28.71 and 99.76% for DPPH and ABTS^**+.**^ inhibition, respectively. The scavenging rates of ESCH to DPPH and ABTS were 38.93 and 91.89%, respectively. The scavenging effect of ABTS was more obvious than that of DPPH in the range of 0.1–0.8 mg/mL. In Figure 4B, F3 clearly had a stronger ability to scavenge ABTS radicals than ESCH. However, in the DPPH radical scavenging experiments (Figure 4A), the opposite results were found.

### Cytokines and MAPK Pathway Analysis

**Figure 5:**
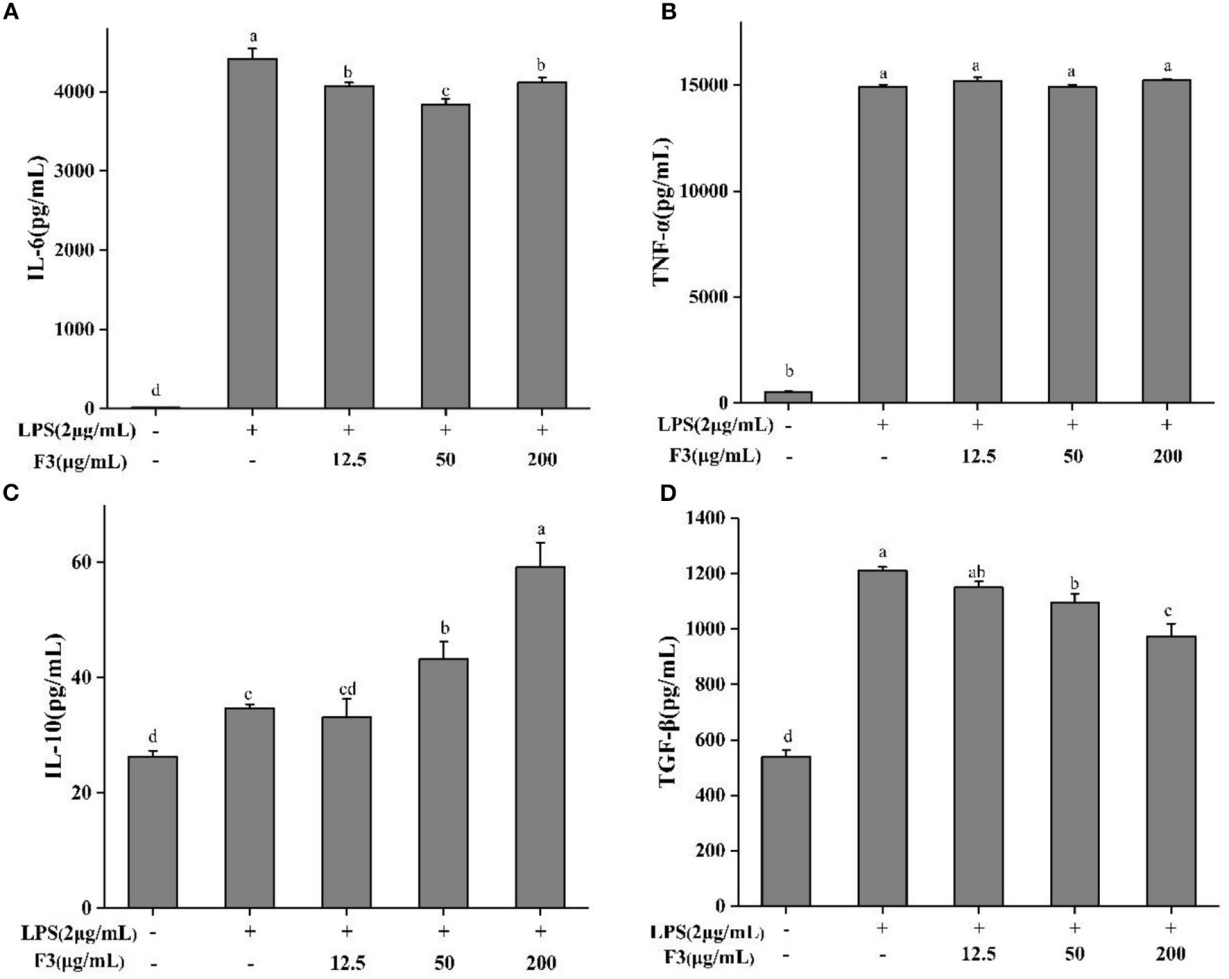
The cytokines IL-6 **(A)**, TNE-α **(B)**, IL-10 **(C)**, and TGF-β **(D)** of pretreatment with F3 in RAW264.7 macrophage. The results were expressed as means ± SD (*n* = 3), where lowercase letters indicate significantly different at *p* < 0.05.

As IL-6, TNF-α, TGF-β, and IL-10 are produced mainly by macrophages, the anti-inflammatory effect of F3 is assessed by measuring these pro- and anti-inflammatory cytokines. F3 pre-treatment decreased the level of IL-6 but not TNF-α (Figures 5A,B). At the concentration of 50 μg/mL, the inhibition of IL-6 was 13.07%. Anti-inflammatory cytokines TGF-β and IL-10 showed an opposite trend (Figures 5C,D). As the concentration of F3 increased, the yield of TGF-β reduced 4.94–19.57%, while the level of IL-10 increased 4.6–70.54% (compared with LPS-induced group).

**Figure 6:**
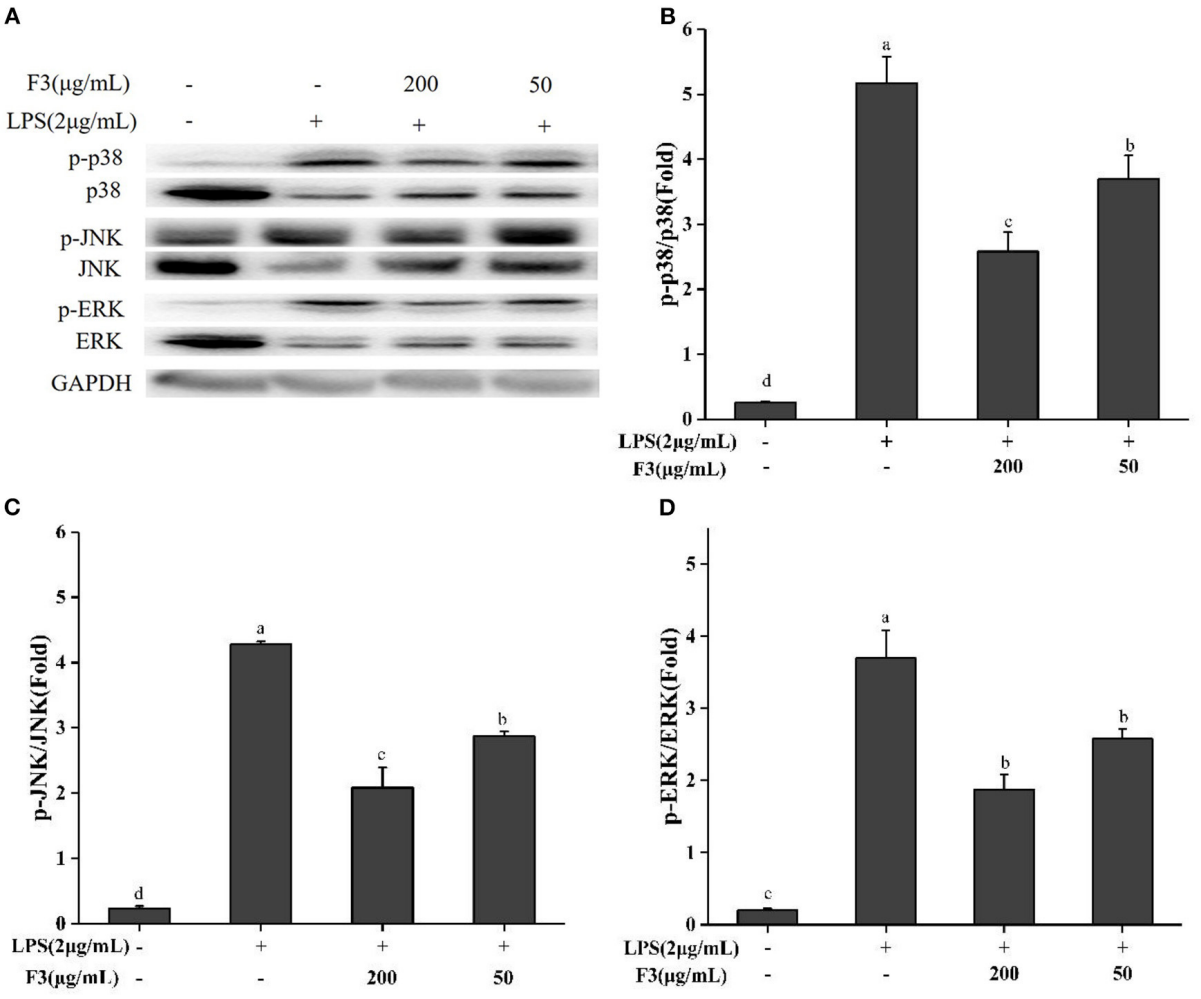
Effects of F3 on the MAPKs proteins in RAW264.7 cells. The levels of p-p38, p38, p-JNK, JNK, p-ERK, and ERK **(A)**. Fold of p-p38/p38 **(B)**, p-JNK/JNK **(C)**, and p-ERK/ERK **(D)**. The results were expressed as means ± SD (*n* = 3), where lowercase letters indicate significantly different at *p* < 0.05.

Previous studies illustrated that chronic inflammatory diseases activate MAPK family proteins (p38, ERK, JNK) (38). Bioactive peptides usually act by reducing the phosphorylation of these proteins. To explore the anti-inflammatory mechanism, the effect of F3 on MAPK signal pathway was investigated by Western blotting. As shown in Figure 6, F3 downregulated the phosphorylation of p38, ERK, and JNK at 200 μg/mL to 46.31, 43.85, and 41.00%, respectively.

### Peptide Sequence Identification

F3 was analyzed by LC-MS/MS (Table 3). The peptides largely showed strong hydrophobicity, with hydrophobic amino acids at the N-terminus and/or C-terminus, which was consistent with the structure of peptides with anti-inflammatory properties.

**Table 3:** Peptide sequences identified by LC-MS/MS in the F3 from ESCH (results were shown based on the relative intensity of mass spectrum, peptide score, and molecular size).

**Peptide sequence**	**Calculated mass**	**Length**	**Source of species**
LGPY	448.2322	4	Amur sturgeon
YPYPDYSR	1059.4661	8	Amur sturgeon
LGGYP	505.2536	5	Amur sturgeon
RGPPGPT	680.3605	7	Amur sturgeon
YPSY	528.222	4	Amur sturgeon
YSLM	528.2254	4	Amur sturgeon
VGPVGP	524.2958	6	Amur sturgeon
FQGL	463.2431	4	Amur sturgeon
LVVY	492.2948	4	Amur sturgeon
AGPAGLPM	754.3683	8	Amur sturgeon
EGAAGGP	557.2445	7	Amur sturgeon
SSMR	479.2162	4	Amur sturgeon

## Discussion

On a dry basis, the protein content of sturgeon cartilage in this study was 52.73%, which was different from Siberian sturgeon cartilage (49.11%) (39) and cartilage powder of *Acipenser sinensis* (58.72%) (40). The differences in protein content may be the results of the species and separation processing technology (41–43). Due to the high-temperature boiling and selective elimination of cartilage particles before chopping and mixing, part of the protein will be lost, resulting in high water content and low protein content.

It has been reported that peptides with immunoregulatory activity and anti-hyperuricaemic activity were obtained from the cartilage by hot-pressure (3, 23, 44), which indicated that hot-pressure can be used as a convenient method to obtain active substances from cartilage. The liquefaction rate of sturgeon cartilage was 98.10% (120°C, 90 min). It was corresponded to that of chicken breast cartilage under the same processing program (31). Compared with hot-pressure, steam explosion, an instantaneous high-pressure method, produced 75.72% of liquefaction rate (32). It implied that long-term high temperature may be more conducive to improving the liquefaction rate of cartilage. In general, a high DH is usually related to the complete hydrolysis of protein, which can produce more peptides with smaller molecular weights. The DH of the cartilage hydrolysates was higher than that of Alaskan pollock (DH 16.87%) hydrolysed by trypsin for 4 h and 50 min (44) and *Rastrelliger kanagurta* (DH 18.1%) hydrolysed by papain for 6 h (45). Lin et al. (24) found that trypsin, alkaline protease and papain had better peptide extraction rates than neutral protease and flavor protease. However, considering the high cost of alkaline protease and the competitive inhibition of enzyme kinetics, double enzyme hydrolysis was used to obtain products with a higher DH in shorter time. Compared to the hydrolysates (DH > 25%) containing 61.62 ± 2.78% low molecular weight peptide (<1 kDa) obtained by alkaline protease hydrolysis for 9 h (24), the content of low molecular weight peptide in sturgeon cartilage was as high as 94.71%, which may mainly because of the hot-pressure (31). It makes the protein migrate into solution and unfold more thoroughly, finally exposing more enzymatic hydrolysis sites. Therefore, hot-pressure combined with double enzyme hydrolysis can be an effective method to promote the release of small molecular peptides from cartilage.

Agarose-gel electrophoresis is used to detect the residue of chondroitin sulfate (46, 47). Chondroitin sulfate usually exists in the form of proteoglycan. It is easily soluble in water and insoluble in organic reagents such as ethanol. In Figure 1A, bands with chondroitin sulfate occurred in components of 75% ethanol-soluble, ethanol-precipitated and > 10 kDa. The difference in content of chondroitin sulfate between two enzymatic hydrolysis methods may result from the higher enzymatic hydrolysis intensity in double enzymatic hydrolysis than single enzyme hydrolysis, which would lead to the differences of the content and properties of proteins/peptides linked to chondroitin sulfate. As the concentration of ethanol increased, the solubility of the chondroitin sulfate further decreased. Therefore, no chondroitin sulfate was detected in 85% ethanol extracted products. Chondroitin sulfate from different sources has different molecular weights. The molecular weight of chondroitin sulfate isolated from chicken, bony fishes and cartilaginous fish were 30–37.18 kDa (32, 33), 13.46–48.68 kDa (46, 47), and 50–70 kDa (48), respectively. It was found that the molecular weight of chondroitin sulfate obtained in sturgeon was at least 10 kDa, which was consistent with previous reports. Overall, 85% ethanol-extracted method can be used to obtain ethanol-soluble component without chondroitin sulfate. It has been reported that chondroitin sulfate extracted from the cartilage of smooth-hound, corb (*Sciaena umbra*) and sturgeon (*Acipenser sinensis*) had the characteristic absorption peaks of polysaccharides at 200, 210–215, and 206 nm in UV spectrum, respectively (46, 48, 49). However, ESCH did not show an absorption peak at 200 nm (Figure 1A), which confirmed the electrophoresis result (Figure 1B). Due to the low content of phenylalanine, tryptophan and tyrosine, there was no obvious absorption peak at 280 nm, while the maximum UV absorption peak of collagen was mainly at 230 nm (39, 50). Figure 1B showed that there was no complete collagen structure in ESCH but peptide. It conformed to the results of amide band appearing in the IR (Figure 1C). No absorption peak characterizing C-O stretching vibration or OH variable angle vibration was found at 1,420–1,375 cm^−1^ (32, 33). It implied that there may be no free acid group. Values of 1,240–925 cm^−1^ are the characteristic absorption of D-glucopyranose ring and sulfate groups (31, 40, 46, 48), which descripts the exitance of carbohydrates in ESCH. The characteristic stretching vibration peak of the axial coordination of C-O-S in ESCH appears at 867.34 cm^−1^. It is close to the position of the characteristic sulfate peak at C4 with an obvious deviation (32, 40). However, high temperature for a long time seemed not influence the chondroitin sulfate structure obviously (33). D-glucopyranose ring and sulfate groups proposed here may not be in the complete chondroitin sulfate. According to the results of agarose gel electrophoresis and spectral analysis, it can be speculated that there were peptides but not chondroitin sulfate in ESCH, although the structure of polysaccharides after degradation could exist in the form of glycopeptides.

Glycine is the most important amino acid in collagen. Except for the first 14 amino acid residues at the N-terminus and the first 10 amino acid residues at the C-terminus, glycine and proline usually form repetitive domains of the Gly-X-Y conservative sequence, where X is proline and Y is any amino acid (51). The high content of glycine, proline and alanine in ESCH was similar to the composition of collagen from Siberian sturgeon (39) and silvertip shark cartilage (50). However, the content of glycine was lower than that in these collagen (326.8–327.5 residues/1,000 residues, 319–326 residues/1,000 residues). Interestingly, ESCH illustrated high content of aromatic amino acids than collagen. This result may be related to the amino acid sequence of peptide and the existence of non-collagen (23). The hydroxyproline content in ESCH was consistent to the type II gelatine of silvertip shark cartilage but lower than the collagen in Siberian sturgeon cartilage (94.7–96.2 residues/1,000 residues) (39, 50). This may be the consequence of body temperature and seasonal variation (50). It was reported that the collagen in cartilage of cartilaginous fishes (including shark, stingray and ray) was composed of type II collagen ([α 1 (II)] 3) and type I collagen ([α 1 (I)] 2 α 2 (I)) at a ratio of 2:1 (50–53). The collagen of Siberian sturgeon cartilage was also confirmed to be a mixture of two kinds of collagen (39). Therefore, the high proportion of type II collagen may be another reason resulting in the lower hydroxyproline content. Based on the results of amino acid composition, it is speculated that ESCH may be a mixed hydrolysate of collagen and extracellular matrix protein. In addition, the large amount of hydrophobic amino acids such as phenylalanine, leucine, isoleucine, valine, proline and alanine is likely to benefit from the separation method of 85% ethanol extraction (54).

Macrophages activated by the immune system can release NO in inflammatory sites to repair tissue, while excessive NO can cause various inflammatory diseases (55). ESCH behaved a good NO inhibition in LPS-induced macrophages. It might signify to further explore the anti-inflammatory effect of peptides from sturgeon cartilage. The anti-inflammatory properties of peptides often depend on their structure, including the amino acid composition and sequence of peptides, the types of C-terminal and N-terminal amino acids, charge, hydrophobicity and space structure (15, 56). The enrichment of hydrophobic amino acids in ESCH may be more conducive to the inhibition of NO (16). A study on the anti-inflammatory activity of sturgeon muscle hydrolysates found that fraction with a higher hydrophobic amino acid content showed a stronger NO inhibition ability, and the peptide identified also proved this hypothesis (6, 37). It was found that hydrophobic amino acids at the N-terminus have important significance on anti-inflammatory property (11). Thus, the strong inhibitory effect of ESCH on NO may come from its high content of hydrophobic amino acids. Here, sturgeon cartilage was proved a good source of anti-inflammatory peptides. IR analysis showed that there might be glycopeptides in ESCH. Glycopeptide hydrolysate is reported to perform the effect of inhibiting the inflammatory development (57, 58). The difference of content between sugar and peptide implied that the inhibitory effect of ESCH on NO may mostly come from the active peptide fragment released after enzymatic hydrolysis (27). Nevertheless, the specific anti-inflammatory mechanism of the peptides and whether the glycopeptides in ESCH have anti-inflammatory effects need further research.

Gel filtration chromatography is an effective technology for separating peptides based on molecular weight. It has been widely used in the separation and desalination of mixed components (59, 60). After separated, F3 performed higher NO inhibition than other fractions. Lee et al. (61) found that after molecular weight grading, the lower molecular weight peptide mixtures showed stronger effect. It was consistent with previous results (54, 62). Moreover, compared with other molecular weight components, sturgeon muscle hydrolysis <3 kDa also showed the superior anti-inflammatory activity in LPS-induced macrophages (5). Therefore, because of the high solubility, low viscosity and hypoallergenicity (26, 62–64), the anti-inflammatory activity of the last eluted fraction obtained by Sephadex G-15 may be related to the molecular weight.

Hydrolysates usually contain amino acids or peptides, which can react with free radicals in the form of hydrogen donors or electron donors and convert them into stable products. DPPH and ABTS free radical scavenging rates are often used to evaluate the antioxidant activity of various hydrolysates (65). The hydrophilic free radical ABTS has a higher binding capacity with F3, while the oil-soluble radical DPPH may react with ESCH extracted by ethanol more easily. This result may be due to the differences in the solubility and diffusibility of free radicals (65, 66). In addition, the difference in free radical scavenging capacity may also be attributed to the peptide length and amino acid composition in the hydrolysate (66, 67).

In RAW264.7 macrophages, LPS stimulation can lead to the excessively release of inflammatory cytokines, including the cytokines TNF-α and IL-6 (37, 68). TNF-α and IL-6 usually will promote the inflammation. F3 reduced the production of IL-6, implying the relief of intracellular inflammation. Out of the instinct to protect the organism, the anti-inflammatory cytokines of IL-10 and TGF-β will be induced to block the uncontrollable inflammation (9, 28, 69). Here, F3 may act as a mitogen or polarizing agent to induce macrophages to transform from M1 to M2, increase the secretion of IL-10 and reduce the level of inflammation (Figure 5C). It is worth noting that each cytokine binds to a specific receptor on the cell surface, which will produce a signal cascade that affects cell function. This cascade may stimulate the production of other cytokines, and increase the number of cell surface receptors of other molecules or inhibit the effects of cytokines themselves (70). Therefore, the produce of TGF-β was inhibited may through other signals in inflammatory macrophages (71, 72).

As a classical signal transduction pathway of the inflammatory response, MAPK has been widely reported (12, 56, 68, 73). The modulation of each MAPK protein by inflammatory factors, related proteins and receptors during inflammation is not exactly the same. TNF-α seems to be regulated by p38 and ERK proteins (37, 56, 73). Therefore, there was no significant change in TNF-α levels after F3 treatment, which may be a result from the incomplete inhibition of p38 and ERK phosphorylation. This finding corresponds with the results of TNF-α regulation of macrophages by hazelnut protein-derived peptide and sturgeon muscle peptide (37, 73).

## Conclusion

In conclusion, F3 from ESCH without chondroitin sulfate could alleviated the inflammation in LPS-induced RAW264.7 macrophages possibly by down-regulating MAPK signal pathway. Once stimulated by LPS, macrophages would produce a large of NO and pro-inflammatory cytokines such as IL-6. When exposed to F3, the secretion of IL-6 was decreased while anti-inflammatory cytokine IL-10 increased, indicating the relief of inflammation. In addition, F3 and ESCH showed the antioxidant ability *in vitro*, which may be connected to the inhibition of phosphorylation in MAPKs protein. Nevertheless, further *in vivo* studies are still needed to clarify the significance of the current study.

## Data Availability Statement

The original contributions presented in the study are included in the article/supplementary material, further inquiries can be directed to the corresponding author/s.

## Author Contributions

QC conceptualization, experimentation, data treatment, interpretation, and writing original draft. LY data treatment, interpretation, validation, and reviewing draft. XW data treatment and interpretation. BY and WZ methodology and investigation. WJ conceptualization and supervision. RG conceptualization, data treatment, interpretation, validation, writing, reviewing, editing, and supervision. All authors contributed to the article and approved the submitted version.

## Conflict of Interest

The authors declare that the research was conducted in the absence of any commercial or financial relationships that could be construed as a potential conflict of interest.
